# A balance of noncanonical Semaphorin signaling from the cerebrospinal fluid regulates apical cell dynamics during corticogenesis

**DOI:** 10.1126/sciadv.abo4552

**Published:** 2022-11-18

**Authors:** Katrin Gerstmann, Karine Kindbeiter, Ludovic Telley, Muriel Bozon, Florie Reynaud, Emy Théoulle, Camille Charoy, Denis Jabaudon, Frédéric Moret, Valerie Castellani

**Affiliations:** ^1^ MeLis, CNRS UMR 5284, INSERM U1314, University of Lyon, Université Claude Bernard Lyon 1, Institut NeuroMyoGène, 8 avenue Rockefeller, 69008 Lyon, France.; ^2^ Department of Basic Neuroscience, University of Geneva, 1211 Geneva 4, Switzerland.; ^3^ UCL Institute of Ophthalmology, University College London, London, UK.

## Abstract

During corticogenesis, dynamic regulation of apical adhesion is fundamental to generate correct numbers and cell identities. While radial glial cells (RGCs) maintain basal and apical anchors, basal progenitors and neurons detach and settle at distal positions from the apical border. Whether diffusible signals delivered from the cerebrospinal fluid (CSF) contribute to the regulation of apical adhesion dynamics remains fully unknown. Secreted class 3 Semaphorins (Semas) trigger cell responses via Plexin-Neuropilin (Nrp) membrane receptor complexes. Here, we report that unconventional Sema3-Nrp preformed complexes are delivered by the CSF from sources including the choroid plexus to Plexin-expressing RGCs via their apical endfeet. Through analysis of mutant mouse models and various ex vivo assays mimicking ventricular delivery to RGCs, we found that two different complexes, Sema3B/Nrp2 and Sema3F/Nrp1, exert dual effects on apical endfeet dynamics, nuclei positioning, and RGC progeny. This reveals unexpected balance of CSF-delivered guidance molecules during cortical development.

## Teaser

Sema/Nrp complexes generate pro-apical and anti-apical forces for cortical progenitors.

## INTRODUCTION

During development of the vertebrate central nervous system, cell cycle kinetics and cell fate decisions of neuronal progenitors are precisely orchestrated by complex intrinsic and extrinsic mechanisms. Cortical stem cells are located in the ventricular zone and exhibit a highly polarized morphology with a basal and an apical process that are anchored to the pial basement membrane and the ventricular surface, respectively (*1*). The apical endfeet are in direct contact with the cerebrospinal fluid (CSF), allowing cortical stem cells to receive extrinsic signals from the cerebral ventricles (*2*). During early corticogenesis, apical progenitors divide symmetrically to expand the pool of progenitor cells. However, at the onset of neurogenesis, they divide asymmetrically to generate both proliferative and postmitotic progeny. Apical endfeet are tightly attached to adjacent neighbors via adherens junctions and cells that are committed to differentiation reduce the apical connections to disengage from the apical surface (*3*–*5*). The detachment is essential for further neurogenesis and migration of the differentiated cells (*6*). Apical progenitors are highly dynamic along the apicobasal axis and undergo interkinetic nuclear migration (INM) in synchrony with their cell cycle. The nucleus oscillates from the apical pole where mitosis occurs to a more basal position where S phase is achieved. Apical endfeet, nuclear dynamic, and cell cycle kinetics are considered as major parameters determining the balance between proliferation and neurogenesis, which is crucial for cortical integrity (*7*). Nevertheless, the developmental mechanisms and factors controlling the spatial architecture of apical progenitors and their dynamic are vastly unknown. The apical endfeet are in direct contact with the CSF of the ventricles, from where they receive extrinsic signals (*2*). Recently, it has been discovered that proteins acting as guidance cues for migrating cells and axons also influence neural progenitor proliferation and differentiation (*8*–*10*). Class 3 Semaphorins (Semas) are secreted proteins exerting either repulsive or attractive effects upon binding to transmembrane receptor complexes composed of Neuropilins (Nrps) and Plexins (*11*). We recently observed that the Semaphorin3B (Sema3B) is released into the CSF by the nascent choroid plexus (CP) and the floor plate, where it influences the proliferation and division orientation of progenitor cells in the developing spinal cord (*12*). This broaches the issue whether soluble Semas in the CSF influence cortical progenitor cells as well.

Here, we provide evidence for an unconventional Sema/Nrp signaling pathway that controls the apical endfeet size, nuclear positioning, and cell adhesion of cortical progenitor cells. Our data suggest that class 3 Semas and soluble Nrps are expressed by the embryonic CP and released into the CSF. They form specific complexes that bind to Plexins, which are present on apical progenitor cells. The resulting signaling exerts collaborative efforts in regulating the apical positioning of mitotic nuclei at the ventricular border and the adhesive properties of these cells. Our results indicate that Sema3B/Nrp2 signaling causes pro-apical forces and increases adhesion that reduces the generation of differentiated cells. In turn, Sema3F/Nrp1 signaling exerts anti-apical forces and decreases adhesion needed for setting transient amplifying cells and postmitotic neurons. Together, our results suggest that extrinsic CSF-derived Sema/Nrp complexes are crucial for apical dynamic of neural progenitors and contribute to the proper generation of subsequently differentiated cells in the developing cerebral cortex.

## RESULTS

### Class 3 Semas are expressed by the embryonic choroid plexus and released into the CSF

**Fig. 1.**
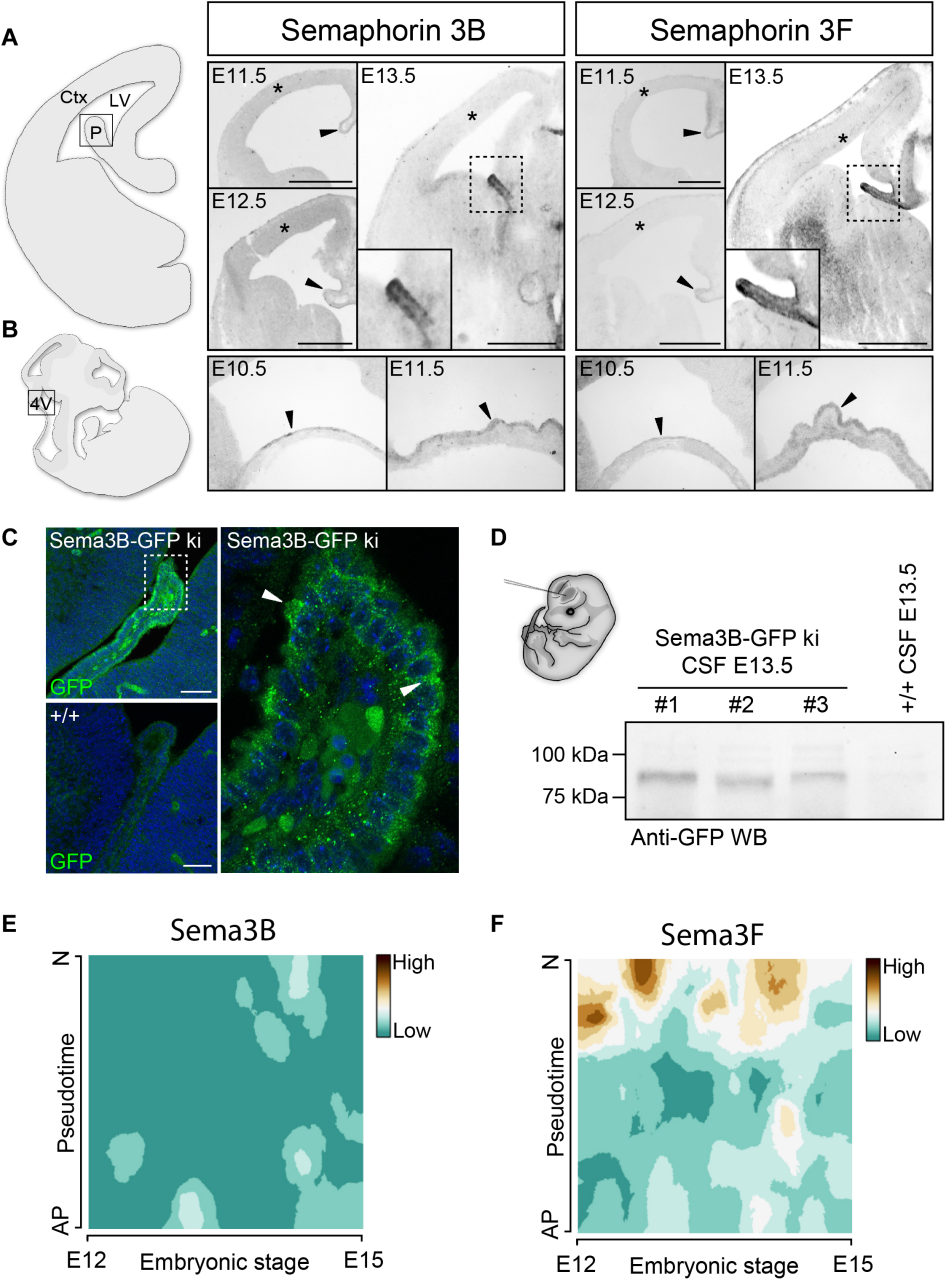
Class 3 Semas are expressed by the developing CP and secreted into the CSF. (**A** and **B**) In situ hybridizations on coronal sections through the telencephalon (A) and sagittal sections of the hindbrain (B) show the mRNA expression of *Sema3B* and *Sema3F* at indicated ages. Arrowheads point to the developing CP. Asterisks indicate the cortical ventricular zone, which is composed of apical progenitors. (**C**) Fluorescence micrographs showing GFP labeling of the nascent CP in Sema3B-GFP ki/ki and WT embryos at E13.5. Arrowheads point to the GFP accumulation at the apical border of the CP. (**D**) GFP immunoblotting on CSF from Sema3B-GFP ki/ki embryos shows the presence of soluble Sema3B-GFP molecules in the CSF. (**E** and **F**) Mapping of *Sema3B* (E) and *Sema3F* (F) mRNA levels obtained from single-cell transcriptome sequencing of embryonic cortical cells between E12 and E15. The “pseudotime” scale indicates the differentiation state of individual cortical cells from an apical progenitor (AP) to a postmitotic neuron (N) deduced from transcriptome analysis. Blue indicates that neither *Sema3B* (E) nor *Sema3F* (F) is expressed by cortical progenitor cells. Scale bars, 500 μm (A) and 100 μm (C). Ctx, cortex; LV, lateral ventricle; P, choroid plexus; 4V, fourth ventricle; WB, Western blot.

To reveal the expression of Sema3s in the developing cerebral cortex, we performed in situ hybridizations on brain sections at different embryonic stages, focusing on Sema3B and Sema3F. We detected both transcripts in the nascent CP of the lateral ventricles starting from embryonic day 11.5 (E11.5), when the formation of this structure is initiated (*13*), with abundant expression of Sema3B and Sema3F mRNA at E13.5 (Fig. 1A). Notably, we observed no signal in the cortical wall. In addition, we detected Sema3B and Sema3F transcripts in the developing CP of the fourth ventricle starting from E10.5 (Fig. 1B). The data suggest that Sema3B and 3F are expressed by the developing CP but not by cortical progenitor cells in the ventricular zone of the telencephalon. These findings were confirmed by single-cell transcriptome analysis of cortical cells using a high-temporal resolution technology allowing fluorescent tagging of isochronic cohorts of newborn ventricular zone (VZ) cells, according to (*14*). Cells from E12 to E15 embryos were analyzed at different states of maturation, starting from apical progenitors to postmitotic neurons. No class 3 Semas were detected in apical progenitors (Fig. 1, E and F). Because no suitable antibodies are available for immunostaining of Sema3B and Sema3F, we took advantage of a *Sema3B-GFP* knock-in (ki) mouse line (*12*) to reveal the endogenous Sema3B expression. In this mouse model, the N terminus of Sema3B is fused to an enhanced green fluorescent protein (GFP), which can be detected with an anti-GFP antibody. GFP immunolabeling confirmed the prominent expression of endogenous Sema3B-GFP in the nascent CP of *Sema3B-GFP* ki embryos at E12.5 (fig. S1A) and E13.5 (Fig. 1C). Moreover, Sema3B-GFP appeared to be concentrated at the apical border of the CP, suggesting that Sema3B is secreted into the CSF. To confirm the presence of class 3 Sema molecules in the CSF, we performed anti-GFP Western blot experiments on CSF samples harvested from the embryonic brain ventricles at E13.5 of *Sema3B-GFP* ki or wild-type (WT) embryos as control. We indeed detected Sema3B-GFP in the CSF of *Sema3B-GFP* ki mice (Fig. 1D). Together, our results suggest that the nascent CP is a main source for Sema3B and Sema3F during cortical development and releases these molecules into the CSF. We thus examined whether cortical progenitor cells express Nrps and Plexins, which form receptor complexes mediating the Sema3 signaling.

### Soluble Nrps are released in the CSF and form complexes with Sema3s that bind to Plexins at apical progenitors

**Fig. 2.**
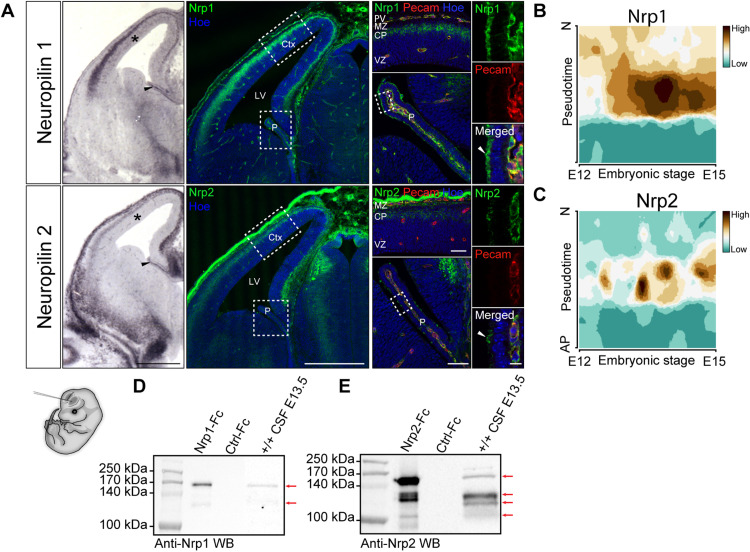
Neuropilins are expressed in the CP, the cortical plate, and other brain-surrounding tissues and released in the CSF. (**A**) In situ hybridization and immunolabeling of Nrp1 and Nrp2 on embryonic brain sections at E13.5. Expression of the class 3 Sema receptors was detected in the nascent CP (black arrowheads), the cortical plate, and meninges. No signal was detected in the cortical ventricular zone (asterisks). Higher magnifications of the cortex and CP colabeled with anti-Pecam highlight Nrp1/2 expression in endothelial cells of the CP and the perineural vascular plexus. The white arrowheads point to the Nrp1/Nrp2 protein signal at the apical border of epithelial cells in the developing CP. High magnifications of the choroid plexi are shown right. (**B** and **C**) Single-cell transcriptome analysis of embryonic cortical cells shows that cortical progenitor cells express neither Nrp1 (B) nor Nrp2 (C) between E12 and E15. Pseudotime describes the differentiation from an apical progenitor (AP) cell to a postmitotic neuron (N). (**D** and **E**) Immunoblotting on embryonic CSF using antibodies against Nrp1 (D) and Nrp2 (E) indicates that soluble forms of both receptors indicated with arrows are released into the brain fluid. Recombinant Nrp1 and Nrp2 ectodomains fused to Fc fragment were used as positive controls of immunodetection. Scale bars, 500 μm (A, left), 50 μm (A, middle), and 10 μm (A, right). CP, cortical plate; MZ, marginal zone; VZ, ventricular zone; M, meninges; PV, perineural vascular plexus.

We first investigated the expression of the Nrp receptors, Nrp1 and Nrp2, using in situ hybridization and immunolabeling (Fig. 2A) on embryonic brain sections at E13.5. With both approaches, we observed Nrp1 and Nrp2 in the cortical plate of the dorsal telencephalon, which reveals Nrp expression in differentiating neurons. Notably, however, we detected neither *Nrp1* nor *Nrp2* mRNA in the cortical ventricular zone, where the proliferating progenitor cells are located (Fig. 2A). These findings were confirmed by real-time functional single-cell transcriptome analysis, indicating that unlike differentiating cortical cells, apical progenitor cells express none of these receptor subunits (Fig. 2, B and C).

**Fig. 3.**
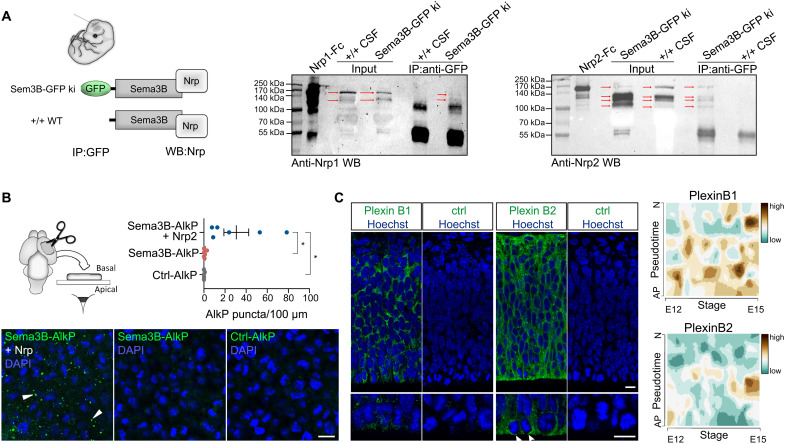
Sema3s and Nrps form complexes in the embryonic CSF that bind to Plexins at the apical surface of radial glia cells. (**A**) Immunoprecipitation (IP) of GFP using CSF of *Sema3B-GFP* ki/ki mice and WT embryos shows prominent binding of Sema3B-GFP with Nrp2 but rare association with Nrp1 in the CSF. (**B**) En face binding assays reveal that exposure of cortical tissue to recombinant Sema3B-AlkP and Nrp2-Fc results in binding of the protein complex to the apical surface. In contrast, Sema3B-AlkP alone and control-AlkP do not bind to the apical surface. The histogram depicts the quantification of AlkP puncta. DAPI, 4′,6-diamidino-2-phenylindole. (**C**) Immunolabeling of PlexinB1 and PlexinB2 on cortical sections at E12.5 shows the expression of these Sema receptors in the developing cortex. Arrowheads point to the accumulation of the PlexinB2 proteins at the apical surface. Single-cell transcriptome analysis of embryonic cortical cells reveals an expression of PlexinB1 and PlexinB2 in apical cortical progenitor cells. Pseudotime describes the differentiation from an apical progenitor (AP) cell to a postmitotic neuron (N) between E12 and E15. Scale bars, 10 μm. Means ± SEM; each dot represents one embryo; paired *t* test, *P < 0.05.

Interestingly, in situ hybridization revealed the expression of both *Nrp1* and *Nrp2* in the developing CP of the lateral ventricle (Fig. 2A). Close examination of Nrp1 staining (Fig. 2A) indicates the presence of Nrp1 in vessel wall cells located in the stroma of choroid plexi, which is reminiscent of expression of Nrp1 in endothelial cells reported in other systems (*15*). Anti-Nrp1 immunostaining detected Nrp1 proteins not only in the stroma of the telencephalic choroid plexi but also at the apical borders of their epithelial cells. In addition, as expected from previous work and shown by our single-cell RNA sequencing data (Fig. 2B), *Nrp1* transcripts were also abundantly detected in the cortical plate and enriched in young postmitotic neurons (*16*). In situ hybridization revealed *Nrp2* mRNA in the stromal compartment of the telencephalic CP, suggesting its expression in vessel walls. We then compared Nrp2 protein expression by immunolabeling of E13.5 *Nrp2^+/+^* and *Nrp2^−/−^* brains and detected Nrp2 at the CP apical border (Fig. 2A and fig. S1C). In addition, we also observed strong Nrp2 expression at both transcript and protein levels in the meninges surrounding the cortex and in cell populations in contact with ventricles in several regions of the diencephalon and the midbrain, particularly the floor plate (fig. S1, C and D). The specificity of Nrp2 labeling was validated by immunolabeling performed on *Nrp2^−/−^* brains (fig. S1C). For Nrp1, we had a *Nrp1^Sema/Sema^* line available that does not allow such validation because the protein lacks Sema3 binding but is still present. Nevertheless, immunolabeling with the secondary antibody alone does not reveal an apical staining of the CP (fig. S1B). These observations indicate that cortical progenitors lack *Nrp1* and *Nrp2* expression, suggesting that if the Sema3s regulate their kinetics, then they may have a nonclassical mechanism of action, with the Nrps delivered by other cellular sources. Given their expression by the CP, we hypothesized that the Nrps and their Sema ligands could be secreted in the CSF and collected by the apical progenitors. This was plausible because for both Nrps, soluble secreted forms generated by ectodomain shedding and encoded by specific splicing variants have previously been reported (*17*). To address whether soluble Nrps are released from the choroid plexi, meninges, and other sources of the neural tube wall, we thought to explore their presence in the CSF. We performed Western blot analysis of the CSF harvested from embryonic brain ventricles of E13.5 WT embryos, using specific antibodies against Nrp1 and Nrp2 characterized in previous studies (*11*). Two Nrp1 and four Nrp2 forms were clearly detected in the CSF (Fig. 2, D and E). Their molecular weights were consistent with Nrp ectodomains resulting from the shedding of integral membrane proteins and with shorter specific soluble isoforms. Thus, both Sema3s and Nrps are released into the embryonic brain ventricles and are available for binding to cortical progenitor cells. We next wondered whether they could form precomplexes in the CSF. Because anti-Sema3B or anti-Sema3F antibodies were not specific enough for biochemical analysis of in vivo samples, coimmunoprecipitation experiments using anti-GFP antibody were performed with CSF from E13.5 embryonic brains of conditional *Sema3B-GFP* ki and from WT embryos as control of specificity. The immunoprecipitation was followed by Western blot against Nrp1 or Nrp2 (Fig. 3A). The results revealed Nrp2/Sema3B complexes in the CSF of *Sema3B-GFP* ki embryos. The sizes of Nrp2 isoforms coimmunoprecipitated with Sema3B are similar to Nrp2 ectodomains of the recombinant Nrp2-Fc control. A weak signal was also detected for the Nrp1/Sema3B condition, suggesting that Nrp1/Sema3B complexes, if present, might be much rare.

To test whether Sema/Nrp complexes bind to the apical surface of cortical progenitor cells, we performed en face binding assays with recombinant proteins. For this approach, hemispheres of E13.5 WT embryos were separated and exposed to recombinant Sema3B fused to alkaline phosphatase (AlkP) together with Nrp2-Fc or control-Fc. After AlkP immunolabeling, the dorsal telencephalon was dissected and flat mounted. Notably, images from the apical surface display the presence of punctate AlkP staining exclusively in the condition where Semas were incubated together with Nrp2-Fc (Fig. 3B).

Nrp recruits signaling co-receptors among Plexin family members (*18*) that trigger intracellular signaling upon Sema3 binding. Apical progenitor cells were reported to express PlexinB1 and PlexinB2, and the deletion of these co-receptors causes impaired cortical neurogenesis (*19*). By single-cell transcriptome analysis, we found transcripts for *PlxnB1* and *PlxnB2* in apical progenitor cells (Fig. 3C and fig. S1E). Immunolabeling of E12.5 embryonic cortical sections against PlexinB1 and PlexinB2 with two different sets of antibodies revealed the presence of PlexinB1 and PlexinB2 in apical progenitors. PlexinB2 was enriched at the feet of cells anchored at the apical border, thus, at expected location to interact with the CSF-derived Sema/Nrp complexes (Fig. 3Cand fig. S1F). Together, these results suggest that CSF-derived Nrps could enable secreted Sema3s to bind to Plexins that are present on the apical surface of cortical progenitor cells.

### CSF-derived Sema/Nrp signaling may be dispensable for self-renewal of cortical stem cells

Previous work by our group has demonstrated that Sema3B delivered in the central canal regulates the division orientation of progenitor cells in the spinal cord (*12*). Therefore, we examined whether Sema/Nrp complexes in the embryonic brain ventricles also contribute to the division orientation of cortical progenitor cells with respect to the apicobasal axis. First of all, we examined the in vivo consequences of loss of Sema/Nrp signaling in *Sema3B* knockout (ko) (*11*), *Sema3F* ko (*20*), *Nrp2* ko (*21*), and *Nrp1^Sema/Sema^* mice, in which the Sema3 binding is selectively disrupted (*22*), respectively. Coronal embryonic brain sections of E12.5 embryos were labeled with antibodies against phospho–histone 3 (PH3), to stain cells undergoing mitosis, and against γ-tubulin to label the spindle poles [according to (*12*)]. Together, our investigations revealed no substantial changes in the division orientation of apical progenitor cells in the developing cerebral cortex of the analyzed transgenic mouse lines (fig. S2A). These results indicate that the CSF-derived proteins do not play a crucial role for the division orientation of apical cortical progenitor cells in the dorsal telencephalon, at least at the examined stage and in the analyzed cortical region.

To more directly assess the functional properties of Sema3B/3F on the self-renewal and multipotency of cortical progenitor cells, we also performed a classical in vitro proliferation experiment, the neurosphere assay. For this approach, cortical single-cell suspensions from embryonic WT brains at E12.5 were prepared and subsequently exposed to different combinations of recombinant Sema-Fc and Nrp-Fc proteins, respectively. After 7 days in culture, characteristic free-floating cell clusters (neurospheres) were formed by proliferating neural stem cells. The size and numbers of the resultant spheres were measured as indicators of whether the added recombinant proteins exert proliferative effects. We observed no significant differences between the conditions, suggesting that the Sema/Nrp signaling does not affect stem cell proliferation in vitro (fig. S2C). These results were consistent with analysis of immunolabeling of embryonic brain sections at E12.5 with specific antibodies against Sox2 (sex-determining region Y-box containing gene 2) that stains apical progenitors and PH3, a marker for mitotic cells, in *Sema3B*^−/−^, *Sema3F*^−/−^, *Nrp1 ^Sema/Sema^*, and *Nrp2*^−/−^ mutant mice. We found no difference in number or distribution of Sox2-positive cells in these mouse mutant lines in comparison to WT littermates (fig. S3A). In addition, no differences were observed in the number of apical PH3-reactive nuclei (fig. S3B) in the analyzed mutant embryos. In addition, no differences were detected in the ratio of mitotic cells in pro-, meta-, and ana/telophase in the mouse mutant lines in comparison to WT littermates (fig. S2D). Similarly, in E13.5 *Nrp1^Sema/Sema^* and *Nrp2* mutant mice, we found no difference in numbers of Sox2-positive cells (fig. S3E). These findings support the conclusion that the CSF-derived Sema3B/3F-Nrp signaling may be dispensable for the expansion and renewal of the embryonic cortical apical progenitor pool.

### Sema/Nrp interactions control the adhesive properties of apical progenitor cells

**Fig. 4.**
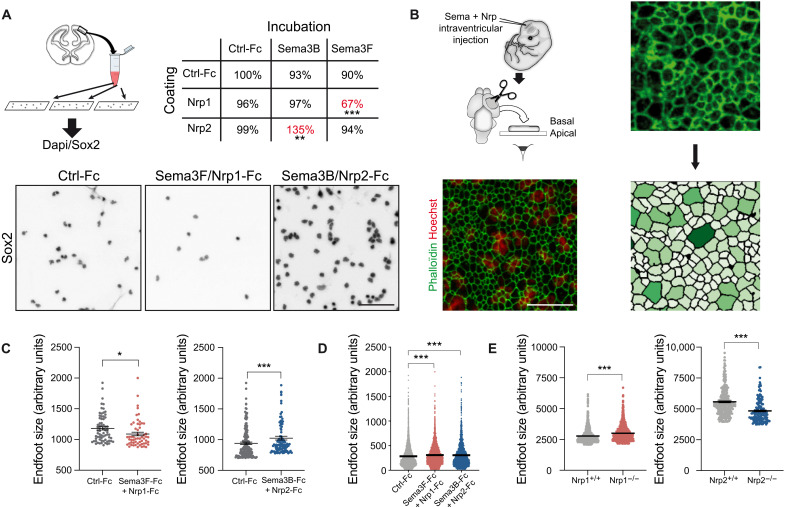
Dual effects of Sema/Nrp complexes on cortical progenitor adhesion and apical endfeet size in the developing cerebral cortex. (**A**) Adhesion assays on coverslips coated with recombinant Nrp-Fc and Sema-Fc molecules in different combinations showed that Sema3F-Fc/Nrp1-Fc inhibited the attachment of Sox2-positive nuclei to the substrate, whereas Sema3B-Fc/Nrp2-Fc promoted cellular attachment. Paired *t* test, **P < 0.01 and ***P < 0.001. (**B**) Injections of recombinant Sema3-Fc, Nrp-Fc, or control-Fc were performed in the lateral ventricles of E12.5 mouse embryos. Cortices were labeled with phalloidin–fluorescein isothiocyanate (FITC) and Hoechst. En face confocal images at the levels of apical endfeet and nuclei of apical progenitors were superimposed. The picture illustrates that mitotic cells have the biggest feet (*32*). The outline of phalloidin-labeled endfeet was detected automatically and the size was measured. (**C**) Quantitative analysis of the apical endfeet area. Phalloidin-labeled apical endfeet were assorted according to their size, and the largest apical endfeet were plotted. Exposure to Sema3B/Nrp2-Fc results in an augmentation of the 2% largest apical endfeet in comparison to control-Fc. In contrast, Sema3-Fc/Nrp1-Fc conditions decrease the size of the 5% largest apical endfeet. KS test, *P < 0.05 and ***P < 0.001. (**D**) Totality of analyzed endfeet after intraventricular injection of control-Fc, Sema3-Fc/Nrp1-Fc, or Sema3B/Nrp2-Fc. Means ± SEM; each dot represents one RGC (*n* = 2 embryos); KS test, ***P < 0.001. (**E**) Analysis of endfeet size in *Nrp1* and *Nrp2* mutant mice. Apical endfeet were labeled with phalloidin-FITC and assorted according to their size, and the 5% largest apical endfeet was plotted. Means ± SEM; each dot represents one RGC (Nrp1, *n* = 4 embryos per condition; Nrp2^+/+^, *n* = 4 embryos; Nrp2^−/−^, *n* = 2 embryos); KS test, ***P < 0.001. Scale bars, 100 μm (A) and 5 μm (B).

Nrps were initially characterized as membrane proteins mediating adhesion, and several studies have highlighted interplays between Semas and Nrps in cell adhesion in various contexts (*23*, *24*). Considering the accessibility of apical adhesion points of apical progenitor cells to CSF-derived cues, we thus hypothesized that the Sema/Nrp signaling could be implicated in the regulation of progenitor apical attachment and positioning. To investigate Sema/Nrp-mediated adhesion in cortical progenitor cells in vitro, we prepared cortical single cells of E12.5 cortices and cultured them on coverslips that were coated with recombinant Nrp-Fc proteins or control-Fc, in the absence of other coating substrate (Fig. 4A). Recombinant Sema-Fc proteins or control-Fc were added to the culture medium. Several cortical cell types may adhere to the substrates, including differentiated neurons. Thus, we use the Sox2 marker to focus on undifferentiated stem cells. We found that exposure to recombinant Sema3F-Fc on Nrp1-Fc–coated coverslips for 3 hours resulted in significantly less Sox2-positive cells binding to the surface. Conversely, Sema3B-Fc application in the Nrp2-Fc–coated condition resulted in an increased number of bound cells (Fig. 4A). In contrast, in neither Sema3B-Fc nor Sema3F-Fc application on control-Fc substrate nor control-Fc application on Nrp1-Fc– or Nrp2-Fc–coated substrates had an effect. Thus, Nrp2/Sema3B complexes may form complexes that increase attachment of apical progenitors, whereas, in contrast, Nrp1/Sema3F complexes act as a nonadherent substrate.

Throughout the neurogenesis period, apical endfeet undergo constant remodeling as radial glial cells (RGCs) divide, newly born cells with RGC fate reestablish apical adhesion, and daughter cells committed to differentiation detach from the apical side and adjacent neighbors. In the in vivo context of an apical delivery, our findings suggested that both CSF-derived complexes could regulate apical anchor dynamics of cortical progenitor cells in an opposite manner. To test this possibility, we created a gain-of-function approach. We isolated E12.5 WT embryos and performed intraventricular injections of either Sema3B/Nrp2-Fc or Sema3F/Nrp1-Fc proteins. Thirty minutes later, cortical tissue was dissected out and their apical surface was imaged following phalloidin/Hoechst staining. We measured the area of apical endfeet and plotted the areas according to endfeet size. We found significant differences between conditions in the general distribution profiles of apical endfeet sizes (Fig. 4D). We focused on the largest endfeet representing the RGC fraction on the ventricular side to compare their size in the different experimental conditions, as rounding of mitotic cells results in an increase of endfeet area (*25*). We found that intraventricular injections of Sema3B/Nrp2 or Sema3F/Nrp1 dually regulated the largest apical endfeet, enlarging and reducing their size, respectively (Fig. 4, B and C). Using similar procedure, we then analyzed endfeet size of apical cells in E12.5 *Nrp1* and *Nrp2* mouse lines. In *Nrp1*^−/−^ mutant embryos, we found an increase in size of the 5% largest apical endfeet, compared to their control littermates. Conversely, in *Nrp2*^−/−^ mutant embryos, the size of the 5% largest apical endfeet was significantly decreased (Fig. 4E). These results were thus fully consistent with our gain-of-function approach.

Next, we studied whether the Sema3B and Sema3F signaling effects on the largest apical endfeet could be exerted by regulating adherens junctions of apical progenitors. We analyzed the intensity of N-cadherin and β-catenin in *Nrp1* and *Nrp2* mutant embryos and their control littermates at two scales: first, along the apical border, thus encompassing the endfeet of a few mitotic cells and a vast majority of interphase cells, and second, specifically on the adherens junctions of apical mitotic cells. For both types of analysis, we found no significant difference between the mutants and their littermate control. We only noted a very subtle difference of β-catenin intensity in *Nrp2* mutants. This suggests that the dual Sema signaling might not primarily affect adherens junctions of apical dividing cells (fig. S4).

### Sema/Nrp signaling modulates the positioning of mitotic progenitors

**Fig. 5.**
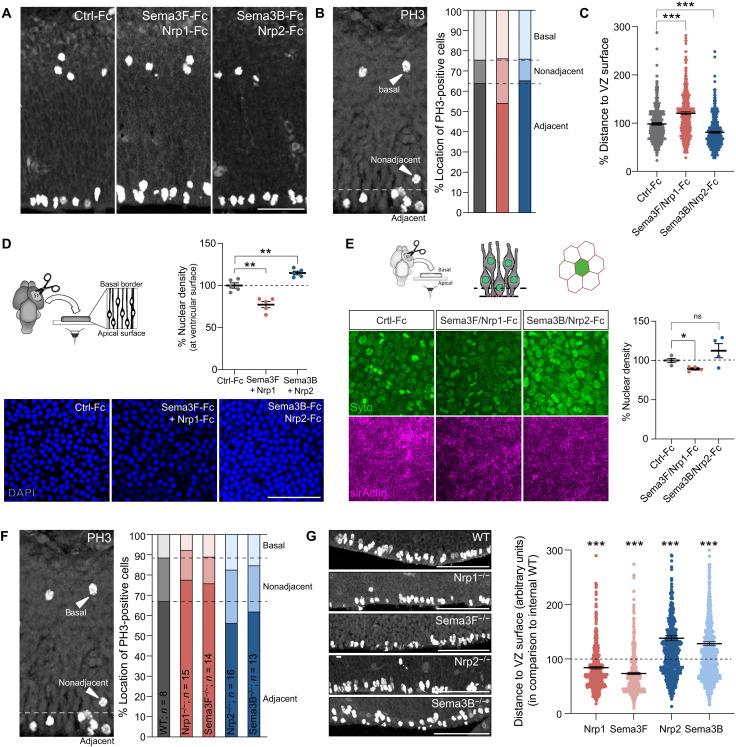
Apically delivered Sema/Nrp complexes regulate the positioning of mitotic nuclei. (**A**) Anti-PH3 immunostaining of E12.5 sections following injections of recombinant Sema3/Nrp-Fc or control-Fc (**B**) PH3-positive nuclei was classified as ventricular, displaced, or basal, according to their position in the cortical wall. (**C**) Analysis of the distance of apical PH3-positive nuclei relatively to the ventricular surface. Means ± SEM; each dot represents one RGC (*n* = 2 embryos); KS test, ***P < 0.001. (**D**) Cortices were apposed to coverslips coated with recombinant Sema3/Nrp-Fc, fixed, and nuclei labeled with Hoechst. The most apical nuclei were imaged with a confocal microscope and counted. Means ± SEM; each dot represents one embryo; paired *t* test, **P < 0.01. (**E**) Cortices were apposed to coverslips coated with recombinant Sema3/Nrp for 30 min. Apical SYTO16-labeled nuclei were live-imaged with confocal microscope in “en face” configuration. The position of the acquisition level with respect to *Z* axis (dotted line) was calibrated relatively to SiR-actin–positive apical endfeet. Less nuclei were present on the apical surface of cortices exposed to Sema3F/Nrp1-Fc. In turn, exposure to Sema3B/Nrp2-Fc resulted in more nuclei present on the apical surface. These differences are reflected in the intensity of the SYTO16 signal. ns, not significant. (**F**) Anti-PH3 immunostaining of cortex sections of E12.5 of *Sema3* and *Nrp* mutants in respect to control littermates. (**G**) Quantification of the relative distance between apical PH3-positive nuclei and the ventricular surface. Means ± SEM; each dot represents one RGC (*n* = 3 embryos); KS test, ***P < 0.001. Scale bars, 100 μm (A, D, and G).

It has already been described that inhibition of apical adhesion results in increased numbers of mitotic nuclei that are mislocated at distance from the ventricular border (*26*, *27*). Delocalization of nuclei in S, G_1_, and G_2_ phases during the INM allows mitotic nuclei to get access to sufficient space at the apical pole (*28*). Similarly, enlargement and retraction of apical endfeet could facilitate apical positioning of mitotic nuclei within RGCs. To examine this possibility, we used our gain-of-function paradigm and assessed whether Sema/Nrp intraventricular injection affects nuclear positioning at the apical surface. After 30 min in vivo, brains were fixed, sectioned, and labeled against PH3. We defined three classes of mitotic nuclei according to their position in the cortical wall [adapted from (*26*)]: adjacent nuclei close to the ventricular surface, nonadjacent nuclei that were more than one cell diameter away from the ventricular border, and basal nuclei that represent intermediate cells (Fig. 5, A and B). Injection of control-Fc resulted in 63.92 ± 1.45% of nuclei adjacent to the lateral surface and 11.68 ± 0.94% of nonadjacent nuclei. Gain of Nrp1-Fc/Sema3F-Fc resulted in increased numbers of nonadjacent nuclei from the lateral surface. We quantified 54.41 ± 2.99% of nuclei adjacent to the lateral surface and 23.21 ± 2.94% of nonadjacent nuclei. The average distance of ventricular nuclei was also increased by 20.52 ± 2.59%. Injection of Nrp2-Fc and Sema3B-Fc had no impact on the radial positioning of the PH3-reactive cells. We detected 65.33 ± 2.12% (*P* = 0.59) nuclei directly next to the lateral surface and 10.64 ± 1.66% (*P* = 0.6) of nonadjacent nuclei. However, the distance of ventricular nuclei was significantly decreased by 18.91 ± 2.43% in comparison to the control-Fc (Fig. 5C). Thus, overall, apical Sema3B/Nrp2 and Sema3F/Nrp1 signaling oppositely affected the apical position of mitotic nuclei, with effects fully consistent with the nature of the regulation that they exerted on the morphology of apical endfeet.

To further validate these findings, we designed an ex vivo experimental paradigm to study whether Sema/Nrp signaling could induce rapid changing of apical nuclei positioning. Cortical tissue was dissected out, to deprive the apical nuclei from access to CSF-derived signals. We then incubated the tissue for 1 hours in vitro (hiv) with the apical surface apposed to coverslips coated with Sema3F-Fc/Nrp1-Fc, Sema3B-Fc/Nrp2-Fc, or control-Fc, respectively. Postfixation staining of F-actin allowed us to visualize the apical endfeet and to control the integrity of the tissue. We quantified the apical density of nuclei (Fig. 5D). In the Sema3F-Fc/Nrp1-Fc condition, the proportion of nuclei on the apical surface was decreased by 24.17 ± 3.65% in comparison to control-Fc. In contrast, in the Sema3B-Fc/Nrp2-Fc condition, it was significantly increased by 13.36 ± 2.17% compared to control-Fc.

In parallel, we developed an en face live imaging of cortical tissue to monitor the dynamics of nuclei exposed to Sema/Nrp molecules. Embryonic cortices were dissected and the ventricular side apposed to glass-bottom dishes coated with Sema3F-Fc/Nrp1-Fc, Sema3B-Fc/Nrp2-Fc, or control-Fc, respectively. The brains were incubated with SYTO16 for nuclear staining and SiR-actin to visualize the apical border (Fig. 5E). Consistent with our previous experiments, we observed differences in nuclear density at the apical side. In the Sema3F-Fc/Nrp1-Fc condition, numbers of apical nuclei were significantly decreased by 10.64 ± 1.51% compared to control-Fc. In contrast, tissue apposed to Sema3B-Fc/Nrp2-Fc revealed a non-significant trend toward the increase of apical nuclei by 12.45 ± 8.98% (*P* = 0.23), once more suggesting a role of Sema/Nrp interactions on the dynamics of apical mitotic nuclei positioning.

Next, we studied whether the invalidation of Semas and Nrps in vivo affects the apical location of mitotic nuclei. We performed an antibody labeling against PH3 in E12.5 embryonic cortical slices of the different mutant mouse lines. The overall number of apical PH3-positive nuclei is not altered in the absence of Sema/Nrp molecules (fig. S3B). The results revealed more mitotic nuclei next to the ventricular surface in *Sema3F* ko and *Nrp1^Sema/Sema^* mice and less nonadjacent nuclei (Fig. 5F). In WT embryos, we quantified 67.02 ± 2.61% adjacent versus 21.38 ± 2.55% nonadjacent PH3-positive cells. In contrast, *Nrp1^Sema/Sema^* mice had 77.5 ± 1.98% adjacent versus 14.51 ± 1.86% nonadjacent nuclei, and *Sema3F* ko mice had 75.42 ± 1.23% (*P* = 0.14) adjacent versus 13.05 ± 1.45% nonadjacent nuclei. Oppositely, mitotic nuclei in *Sema3B* and *Nrp2* ko cortices were radially more scattered in the cortical wall than in the WT, with augmented numbers of basal PH3-positive cells. We quantified 56.19 ± 2.25% (*P* = 0.11) adjacent and 26.57 ± 1.92% (*P* = 0.59) nonadjacent nuclei in *Nrp2* ko mice and 61.89 ± 2.68% adjacent and 22.86 ± 1.99% nonadjacent nuclei in Sema3B-deficient embryos.

In addition, we measured the distance between mitotic nuclei in the ventricular zone and the ventricular surface (Fig. 5G). In comparison to WT littermates, the mitotic nuclei in the VZ of *Sema3F*^−/−^ and *Nrp1^Sema/Sema^* were closer to the ventricular surface for about 26.52 ± 2.258% and 15.96 ± 2.33%, respectively. In contrast, the more distant mitotic nuclei in *Sema3B*^−/−^ and *Nrp2*^−/−^ cortices were reflected in an extended distance to the ventricular surface. Sema3B-deficient embryos exhibited an increased distance of PH3-positive nuclei of about 28.1 ± 3.4% and *Nrp2*-deficient embryos of 38 ± 4.71%. Together, these data indicate that the position of apical progenitor nuclei during mitosis is influenced by CSF-derived signals and support that two Sema/Nrp signaling may exert collaborative effort in balancing pro- and anti-apical forces to regulate nuclear position of dividing apical progenitor cells.

### The generation of intermediate precursors and postmitotic neurons is modulated by CSF-derived Semas/Nrps

**Fig. 6.**
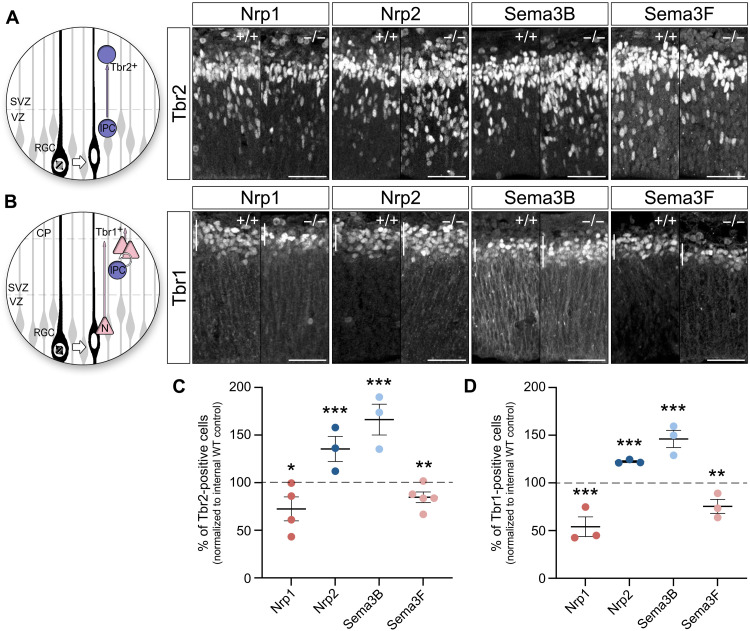
Sema/Nrp complexes regulate the generation of intermediate precursors and postmitotic cortical neurons. (**A**) Tbr2-positive intermediate progenitors delaminate from the apical surface and translocate their soma into the SVZ. Microphotographs illustrate Tbr2-positive nuclei in WT, *Nrp1^Sema/Sema^*, *Nrp2*^−/−^, *Sema3B*^−/−^, and *Sema3F*^−/−^ embryonic cortices. (**B**) During early neurogenesis, Tbr1-positive postmitotic neurons are generated directly by radial glia cells or indirectly by intermediate progenitor cells. Microphotographs illustrate Tbr1-positive nuclei in *Nrp1^Sema/Sema^*, *Nrp2*^−/−^, *Sema3B*^−/−^, and *Sema3F*^−/−^ embryonic cortices. (**C**) The plot shows that *Sema3F^−/−^* and *Nrp1^Sema/Sema^* cortices have less Tbr2-positive cells in comparison to the WT littermates at E12.5. In contrast, Sema3B and Nrp2 ko embryos show significant augmented numbers of intermediate progenitor cells. (**D**) The plot shows that *Sema3F*^−/−^ and *Nrp1^Sema/Sema^* mice exhibit reduced numbers of Tbr1-positive neurons in the cortical plate at E12.5. In contrast, Tbr1-reactive cells are increased in *Sema3B* and *Nrp2* ko mice in comparison to WT littermates. Scale bars, 50 μm. CP, cortical plate; VZ, ventricular zone; SVZ, subventricular zone. Means ± SEM; each dot represents one embryo; paired *t* test, *P < 0.05, **P < 0.01, and ***P < 0.001.

The regulation of apical adhesion and nuclear positioning is particularly important during asymmetric cell division, when one daughter cell remains attached in the neuroepithelium as an apical progenitor, whereas the other one disengages from the ventricular surface to differentiate into a neuron or an intermediate progenitor, which then gives rise to two neurons. We thus analyzed whether deletion of *Semas* and *Nrps* affects the number and distribution of intermediate precursor cells (IPCs). Embryonic coronal brain slices of E12.5 WT and mutant embryos were stained with an antibody against Tbr2 (T-brain 2), a specific molecular marker to identify transient amplifying cells (*29*–*31*), and Tbr2-positive cells were counted (Fig. 6, A and C). We found decreased numbers of IPCs in *Sema3F*^−/−^ and *Nrp1^Sema/Sema^* mice in comparison to WT littermates of about 15.28 ± 5.58% and 27.52 ± 12.56%, respectively. In contrast, *Sema3B* and *Nrp2* ko embryos showed significant augmented numbers of transient amplifying cells. *Sema3B*^−/−^ mice reveal 66.3 ± 16.18% more IPCs, and in *Nrp2*-deficient mice, the numbers were increased for about 35.4 ± 13.22%. Transient amplifying cells are the most prominent precursors during middle and late neurogenesis (*32*), and they generate the majority of postmitotic neurons of the cerebral cortex (*33*, *34*). However, during neurogenesis, postmitotic neurons can be produced directly by apical progenitor cells (*34*). Therefore, we further investigated the numbers of postmitotic neurons generated in the different mouse lines at E12.5, using an antibody against Tbr1 that is specifically expressed in early-born neurons and of layer 6 (*29*, *31*). In agreement with our previous findings of the intermediate progenitor pool, *Sema3F* ^−/−^ and *Nrp1^Sema/Sema^* embryos exhibited lower numbers of postmitotic neurons at E12.5 (Fig. 6, B and D). In comparison to WT littermates, decrease by 24.56 ± 7.39% was observed in *Sema3F*^−/−^ embryos and by 45.92 ± 10.35% in *Nrp1^Sema/Sema^* embryos. In contrast, the number of postmitotic neurons was increased by 46.1 ± 9.03% and 22.4 ± 1.07% in Sema3B^−/−^ and Nrp2^−/−^ embryos, respectively. These results indicate that the pro-apical Sema3B/Nrp2 signaling inhibits the generation of Tbr1-positive neurons and Tbr2-positive intermediate progenitor cells, whereas the anti-apical Sema3F/Nrp1 signaling has an opposite effect, promoting the generation of Tbr2- and Tbr1-positive cells.

### GSK3 mediates the effect of Sema3B/Nrp2 and Sema3F/Nrp1 signaling on apical nuclei position

Several studies reported that glycogen synthase kinases (GSK3s) act downstream of the Semas in the context of axon guidance (*35*, *36*). In addition, we found in previous work that Sema3B regulates the orientation of spinal cord progenitor divisions via GSK3 signaling. GSK3 activity has further been shown to be indispensable for proper dynamics of apical glia cell scaffold in the developing cerebral cortex (*37*), and GSK3 proteins were also proposed to be essential regulators of proliferation and differentiation during corticogenesis (*38*). In light of these contributions, we wondered whether the Sema signaling regulation of apical progenitor positioning is mediated by GSK3s.

To explore a contribution of GSK3, we took advantage of our in vivo paradigm of intraventricular injections. GSK3 inhibitor SB216763 (50 μM) was injected in the lateral ventricles of E12.5 embryos, combined or not with Sema3B/Nrp2-Fc or Sema3F/Nrp1-Fc for 45 min. We analyzed apical nuclear positions in transverse sections stained with phalloidin and Hoechst, as previously described (fig. S5A). We observed that when applied alone, the GSK3 inhibitor had no significant effect compared to vehicle control. In contrast, we found that GSK3 inhibitor prevented Sema3B/Nrp2-Fc to exert their pro-apical effect on nuclei position while not significantly affecting the anti-apical effect exerted by Sema3F/Nrp1-Fc (fig. S5B). Thus, these data suggested that the dual signaling activated by the apical Semas is differentially regulated, with GSK3 specifically contributing to the functions of Sema3B/Nrp2 complexes.

## DISCUSSION

### CSF Sema3s have a noncanonical mode of action

Our study provides evidence for an unconventional Sema/Nrp signaling mechanism in which both ligands and receptors are synthesized by the nascent CP and released into the CSF. Sema3s represent a class of secreted molecules with already reported expression in the developing CP for Sema3B and Sema3F (*12*, *39*). Unexpectedly, we found that Nrp1 and Nrp2, which are, apart for Sema3E, obligatory components of Sema3 receptors, are not expressed by apical progenitors. Rather, they are produced by the nascent CP, the floor plate, and meninges, tissues known to secrete molecules in the CSF. Soluble forms of Nrps have been previously reported in physiological and pathological contexts. In particular, Nrp1 in the CSF was proposed to be associated with Alzheimer’s disease and aging in humans (*40*, *41*). Soluble forms of Nrps in the CSF could arise from splice variants encoding forms devoid of transmembrane domains, similar to those previously described for both Nrp1 and Nrp2 (*17*, *42*). They could also result from ectodomain shedding (*43*). Up to now, the biological functions of these soluble Nrps have remained elusive. In vitro application of soluble Nrps has various outcomes that might depend on the expression profile of Nrp binding partners and components of downstream signaling cascades in the exposed cells. Hence, soluble Nrps were found to display dominant negative effects on the Sema3 signaling, acting via ligand titration and competition with endogenous Nrps. The Nrp1 extracellular domain was also found to reverse Sema3E/PlxnD1-mediated repulsion to attraction in subiculo-mammillary neurons (*44*). Our findings provide the first evidence that trans-delivery of soluble Nrps can reconstitute Sema3 receptor complexes with dual functions in neural progenitors expressing Plexins only. The conformation of Sema3/Plxn/Nrp complexes has been recently highlighted by crystallography data reporting that Nrp ectodomain is indeed required for stabilizing weak interactions contracted between Sema3 and PlxnA molecules (*45*). These structural features are fully compatible with the mode of presentation of the different components of the ternary complex that we report in the developing cerebral cortex, with the CSF delivering preformed Sema3/Nrp complexes to apical Plxns. We found that apical progenitor cells express Plexins, particularly PlxnB2, which accumulates at the ventricular surface of progenitor cells, the expected location for interactions with CSF-derived Sema3/Nrp complexes. Previous work reported that *PlxnB1/B2* double-mutant mouse embryos exhibit cortical development defects, with decreased proliferation and reduced neuronal production, resulting in cortical thinning (*19*). *PlxnB1* and *PlxnB2* are likely redundant because individual deletions do not strongly affect cortical development (*19*, *46*–*48*). Thus, contributions of PlexinB1/2 in the CSF-derived Sema3B and Sema3F signaling are plausible, although additional studies are needed to validate their functional implication.

### Opposite positive and negative Sema3 signaling act at the apical pole of the developing cortex

We found that two conspicuous combinations of Sema3/Nrp complexes, Sema3B/Nrp2 and Sema3F/Nrp1, exert opposite influences on cortical progenitor adhesion and positioning. Whereas Sema3B/Nrp2 generates pro-apical forces, Sema3F/Nrp1 has opposite effects on apical progenitor cells. Possibly, this dual signaling could act together, setting a balance of forces to adapt adhesion and nucleus position at the apical border. Alternatively, the contribution of each component could change with developmental progression. It is conceivable that during early corticogenesis, the effect of Sema3B/Nrp2 interaction predominates to precisely control apical nuclei positioning. At later stages, Sema3F/Nrp1 signaling might prevail to facilitate apical detachment and differentiation. Double deletion of Sema3B and Sema3F did not normalize the number of TBR2-positive cells at E12.5, rather revealing a phenotype resembling that of the single Sema3F deletion thus possibly reflecting nonstrictly symmetrical contributions (fig. S3, C and D).

Whereas opposite outcomes of Sema3/Nrp/Plxn signaling have been observed in various contexts (*49*), the generation of duality of the effects remains puzzling. First, differences might come from specific conformations of ternary Sema3/Nrp/Plxn complexes, possibly raised not only from binding interactions but also from secondary interfaces formed between three-dimensional domains, reported to regulate the affinity and specificity of modular domain–mediated interactions (*50*). Second, differences could already be prefigured by Sema3/Nrp complexes, since prominent preferential association of Sema3B with Nrp2 rather than Nrp1 was observed in our biochemical analysis of complexes in the CSF of *Sema3B-GFP* ki embryos. Last, Sema3B/Nrp2 complexes and Sema3F/Nrp1 complexes could preferentially associate with distinct Plxn co-receptors, then triggering specific downstream signaling and functional outcomes. This hypothesis is supported by our data showing that GSK3 inhibition affected the effects of Sema3B/Nrp2 but not Sema3F/Nrp1 on cell nuclei position. Deep investigations of the specificities generated by these different architectures of ligand/receptor complexes will allow further understanding the variety of biological outcomes they generate.

### Apical Sema signaling exerts functional effects on cortical RGSs

The apical domain, which is composed of adherens junctions and a cortical actin network, is remodeled during the INM, enlarging when nuclei are close to the ventricular surface and shrinking when they migrate basally (*51*). In vivo, mitotic cells have access to the CSF-delivered signals by their apical endfeet. Our study shows that dual Sema3/Nrp signals delivered from the CSF regulate the morphology of endfeet of apical mitotic cells, which may occur via local regulation of adhesion. These effects were not accompanied by modifications of adherens junctions, since we found no differences of distribution and intensity of N-cadherin and β-catenin markers after genetic removal of Nrp1 and Nrp2. Axon guidance molecules play prominent roles in cell shape and adhesion remodeling accompanying tissue formation and function, acting via various mechanisms. For example, the Sema signaling regulates morphological remodeling of podocytes in the kidney (*52*) and hypothalamic neurons secreting gonadotropin-releasing hormone in the brain (*53*). Beyond adherens junctions, adhesion features also depend on actin cytoskeleton assembly and coupling with transmembrane adhesion proteins, as well as on internal relaxing versus contractile forces generated by molecular motors. Internal contractility within the apical domain is also thought to be an important parameter of progenitor divisions. These are plausible mechanisms of action of the dual Sema signaling.

Moreover, both our loss-of-function and gain-of-function experiments provide evidence that opposite effects of Sema3B/Nrp2 and Sema3F/Nrp1 set the apical position of mitotic nuclei as removal of each counterpart results in pro- or anti-apical nuclei shift. Forces moving the nuclei during the INM require activity of actomyosin molecular motors (*61*). Given the pleiotropic properties of the Semas, it remains to be understood which of adhesion, cell shape remodeling, or nucleus movement is primarily regulated in the context of apical progenitor mitoses and whether all three could be coordinated to ensure proper nuclei position and apical endfeet size and shape. Possibly, the dual apical Sema3 signaling could act by regulating the intracellular machinery moving the nucleus, properties already reported during cell migration in the developing brain (*54*). Alternatively, through coupling of adhesion and internal contractility activities, Sema-mediated regulation of endfeet morphology could enable adaptation of the level of apical crowding and endfeet diameter to position the nucleus of mitotic cells during the INM.

CSF-derived cues were shown to regulate progenitor proliferation and differentiation (*2*, *55*). We found that the generation of neurons and intermediate progenitors is increased in *Sema3B* and *Nrp2* mutants, whereas it is decreased in Sema3F and Nrp1 mutants, thus indicating that these signaling also dually contribute to cell fate determination of RGC progeny.

Beyond mitotic nuclei positioning, modulation of the adhesion of apical endfeet is crucial to the delamination of cells from the ventricular surface and is thought to be an instructive event of the emergence of basal progenitors and of the detachment of newborn neurons (*5*, *6*). In support, manipulations of the Eph/Ephrin signaling in neural progenitor cells were found to alter apical adhesions and to modify nuclei position and cell fate during the neurogenic period (*26*). The dual apical Sema3 signaling could thus modulate the generation of intermediate progenitors and neurons through control of neural progenitor adhesion of apical endfeet.

Several previous works reported release of various axon guidance molecules in the CSF, such as Sema4D, Sema7A, Sema3C, and Slits (*13*, *56*, *57*). Our findings bring the first insights into a likely much broader developmental program orchestrating apical dynamics of cortical progenitors. Moreover, given the great diversity of cortical progenitor shapes and radial anchors that emerged in the primate lineage, whether the contributions of such apically delivered axon guidance molecules have been complexified through evolution is a fascinating question for future investigations (*1*, *58*, *59*).

## MATERIALS AND METHODS

### Animals

All animal procedures were performed in accordance to European Communities Council Directive and approved by French ethical committees. Time-pregnant mice of the following strains were used: WT OF1 (Charles River Laboratories, strain 612), *Sema3B* ko (*11*), *Sema3F* ko (*20*), *Nrp2* ko (*21*), and *Nrp1^Sema/Sema^* mice in which the Sema3 binding is selectively disrupted (*22*). The day of insemination was considered as E0.5. Mice were bred and maintained under standard conditions with access to food and water ad libitum on a 12-hour light/dark cycle.

### Section preparation

For the preparation of time-staged embryonic brains, time-pregnant mice were deeply anesthetized with isoflurane, and the embryos were dissected. Embryonic heads were fixed overnight in 4% paraformaldehyde [PFA; in 1× phosphate-buffered saline (PBS) (pH 7.4)] at 4°C, followed by a sequential sucrose treatment [10, 15, and 30% in 1× PBS (pH 7.4)] as cryoprotection. After freezing in isopentane and dry ice at −40°C, heads were sectioned into 20-μm slices (at −21°C) using a Cryotome HM550 (Microm) and mounted on Superfrost Plus slides (Thermo Fisher Scientific).

### Immunolabeling

Brain slices, cortical preparations, and dissociated single cells were washed in 1× PBS (pH 7.4) with 0.2% Tween 20, followed by blocking (in 4% bovine serum albumin in 1× PBS/0.2% Tween 20) for 2 hours at room temperature (RT) and an incubation with the primary antibody overnight at 4°C. After washing, the secondary antibody was applied for 2 hours at RT. The nuclei were stained for 15 min at RT with Hoechst 33342 (1 ng/ml in H_2_O). The following primary antibodies were used: rabbit anti-GFP (1:100; Invitrogen, A11122), goat anti-Nrp1 (1:100; R&D, AF566), goat anti-Nrp2 (1:100; R&D, AF567), rat anti-PH3 (1:50; Sigma-Aldrich, HT28), mouse anti–γ-tubulin (1:100; Sigma-Aldrich, GTU488), rabbit anti-PlexinA2 (1:25; Santa Cruz Biotechnology, sc-25640), rabbit anti-PlexinB1 (1:100; Abcam, ab39717), rabbit anti-PlexinB1 (1:200; antibodies-online, ABIN749228), rat anti-PlexinB2 (1:100; Invitrogen, 772417), rabbit anti-PlexinB2 (1:200; Proteintech, 10602-1-AP), rabbit anti–alcaline phosphatase (1:300; Gene Hunter, Q301), rabbit anti-Sox2 (1:400; Abcam, 97959), rabbit anti-Tbr1 (1:400; Abcam, ab31940), rabbit anti-Tbr2 (1:400; Abcam, 23345), anti-ZO1 (1:100; Thermo Fisher Scientific, 40-2200), anti–N-cadherin (1:200; M142, Takara), and anti–β-catenin (1:400; Merck, 8E7). The following secondary antibodies were used: donkey anti-goat immunoglobulin G (IgG) Alexa Fluor 488 (1:500; Molecular Probes), donkey anti-goat IgG Alexa Fluor 555 (1:500; Molecular Probes), donkey anti-mouse IgG FP547H (1:500; Interchim), donkey anti-rabbit IgG FP647H (1:500; Interchim), donkey anti-rabbit IgG Alexa Fluor 488 (1:500; Invitrogen), donkey anti-rat IgG Alexa Fluor 488 (1:500; Molecular Probes), and donkey anti-rat IgG FP647H (1:500; Interchim).

### In situ hybridization

Digoxigenin (Dig)–labeled probes against Sema3B, Sema3F, Nrp1, and Nrp2 were used as previously described (*68*). Briefly, sections were fixed for 10 min in 4% PFA [in 1× PBS (pH 7.4)], permeabilized for 10 min in 0.2 M HCl, and acetylated in 0.1 M triethanolamine with 5 mM acetic anhydrid for 15 min. Hybridization was performed overnight at 56°C with a probe concentration of 3 ng/μl. Slides were blocked for 2 to 3 hours, using 2% blocking reagent (Roche), followed by the detection of the Dig-labeled riboprobe with an anti-DIG Fab fragment conjugated with AlkP (1:750; Roche). The colometric reaction was performed using a mixture of 5-bromo-4-chloro-3-indolyl phosphate (Roche) and Nitro blue tetrazolium chloride (Roche).

### Western blotting and immunoprecipitation

CSF was directly denatured for 5 min at 95°C in Laemmli buffer. Cortical tissue was lysed for 45 min on ice in 0.5% NP-40 (Sigma-Aldrich), 0.5% SDS, 150 mM NaCl, 50 mM tris-HCl, 2 mM EDTA, and protease inhibitor cocktail (Roche). For Western blotting, samples were denaturated for 5 min at 95°C in Laemmli buffer. For immunoprecipitation, lysates were incubated at 4°C overnight with rabbit anti-GFP (1:1000; Roche), and pull-down was performed with magnetic protein A beads (Millipore) for 15 min at RT. After several washing steps, proteins were collected in 80 ml of Laemmli buffer, and 20 ml of solution was used for SDS–polyacrylamide gel electrophoresis (10% acrylamide precast mini gels, Bio-Rad). A Trans-Blot Turbo Transfer System (Bio-Rad) was used to transfer the proteins in a nitrocellulose membrane that was blocked in 5% milk powder (in 1× PBS/0.2% Tween 20) for 30 min at RT and incubated with the primary antibody overnight at 4°C. Membranes were washed in 1× PBS/0.2% Tween 20 and incubated with the secondary antibody for 1 hour at RT. For signal detection, ECL Prime was used according to the manufacturer’s instructions (RPN2232, G.E. Healthcare) and the ChemiDoc MP system with applied software (Bio-Rad). The following primary antibodies were used: rabbit anti-GFP (1:5000; Invitrogen, A11122), goat anti-Nrp1 (1:1000; R&D, AF566), and goat anti-Nrp2 (1:1000; R&D, AF567). The secondary antibodies were used: anti-goat horseradish peroxidase (HRP; 1:5000; Sigma-Aldrich, A5420) and anti-rabbit HRP (1:5000; Sigma-Aldrich, A9169).

### Preparation of cortical single cells

Time-pregnant mice were deeply anesthetized with 10% chloral hydrate. Brains of the embryos were dissected in PBS (Invitrogen) supplemented with 0.65% glucose, and cortical tissue was dissected and incubated in PBS with 2.5% trypsin for 17 min at 37°C. Afterward, tissue was dissociated by trituration and filtered through nylon gaze to remove cell aggregates. Cells were seeded on coverslips coated with Laminin (19.5 μg/ml; Sigma-Aldrich) and poly-l-**lysine** (5 μg/ml; Invitrogen) at a density of 300 cells/mm^2^ and incubated at 37°C and 5% CO_2_ in a humid atmosphere in Dulbecco’s modified Eagle’s medium (DMEM; Invitrogen) supplemented with 10% fetal bovine serum (FBS), penicillin (10,000 U/ml), streptomycin (10,000 μg/ml), 0.065% d-glucose, and 0.4 mM l-glutamine. For immunostaining, cells were fixed in 4% PFA [in 1× PBS (pH 7.4)] for 10 min at RT.

### Binding assay

To test the binding of recombinant Sema/Nrp complexes to apical progenitors, we incubated brain hemispheres with recombinant Sema3B-fused to AlkP in the presence or absence of recombinant Nrp1-Fc or Nrp2-Fc (R&D Systems), respectively (10 ng/μl in PBS). After 30 min of incubation at 37°C and 5% CO_2_ in a humid atmosphere in DMEM (Invitrogen), hemispheres were fixed in 4% PFA [in 1× PBS (pH 7.4)] overnight at RT, and immunolabeling was performed as described, followed by dissection of the somatosensory cortex and flat mounting with Mowiol.

### Lateral ventricle injection

Rostral injections of 2 μl of recombinant proteins [Sema3B-Fc/Nrp2-Fc, Sema3F-Fc/Nrp1-Fc, control human IgG1-Fc (R&D) diluted at 1 μg/ml, with 50 μM SB216763 GSK3 inhibitor (Sigma-Aldrich) or dimethyl sulfoxide and Fast Green in PBS] were bilaterally performed with glass micropipettes in telencephalic ventricles of freshly harvested E12.5 mouse embryos. Embryos were then incubated 30 min in DMEM (Invitrogen) supplemented with 10% fetal calf serum, 0.65% glucose, and 0.4 mM glutamine at 37°C and 5% CO_2_.

### En face live cell imaging

Time-pregnant mice were deeply anesthetized with 10% chloral hydrate. Brains of the embryos were dissected in ice-cold PBS (Invitrogen) supplemented with 0.65% glucose. The hemispheres were separated and incubated with SYTO16 (1:1000; Invitrogen) and SiR-actin (1:1000; Spirochrome) in DMEM (Invitrogen) for 20 min on ice. After transferring the hemispheres to ice-cold DMEM, the somatosensory cortex was dissected and placed into glass-bottom dishes (Matec) that were coated with poly-l-lysine (1 μg/ml) and recombinant proteins in different combinations (10 ng each) for 1 hour at 37°C. After incubation for 20 min at RT in 100 μl of culture medium [DMEM (Invitrogen) with 25% Hanks’ balanced salt solution (Invitrogen), 10% FBS, penicillin (10,000 U/ml), streptomycin (10,000 μg/ml), 0.65% d-glucose, and 0.4 mM l-glutamine] supplemented with 0.5% methyl cellulose, compartments were filled with culture medium and 0.1 mM Hepes (Invitrogen) before live cell imaging was performed. Pictures were taken using IQ3 software with multi-positions and *Z*-stack protocols. To reduce exposure time and laser intensity, acquisitions were done using binning 1 × 1.

### Detection and analysis

Pictures of in situ hybridization experiments were taken using a Z1 observer microscope (Zeiss). Pictures of the apical surface or brain slices were taken with an inverted confocal laser-scanning microscope FV1000 (Olympus). En face live cell imaging was performed using the Olympus IX81 microscope equipped with a spinning disk (CSU-X1 5000 rpm, Yokogawa), an Okolab environmental chamber, an electron multiplying charged-coupled device (EMCCD) camera (iXon3 DU-885), and applied software (Andor Technology). Photographs were analyzed with Fiji software (*69*). Data collection and analysis were performed blindly. Analysis of cell division orientation was performed as described in (*12*). Quantifications are represented as means ± SEM, and sample size and statistical significance are indicated in the figure legends. Statistic tests and graphs were performed with GraphPad Prism software. *P < 0.05 was considered as significant, **P < 0.01, and ***P < 0.001. Measures of the endfeet area were performed with Cell Profiler software as in (*60*).

## Notes

View/request a protocol for this paper from *Bio-protocol*.
