# Supplementary material for: A balance of noncanonical Semaphorin signaling from the cerebrospinal fluid regulates apical cell dynamics during corticogenesis
Source: Sci Adv. 2022 Nov 18;8(46):eabo4552. doi: 10.1126/sciadv.abo4552 (PMC9674300; doi:10.1126/sciadv.abo4552)

Science Advances
AAAS

Supplementary Materials for

A balance of noncanonical Semaphorin signaling from the cerebrospinal fluid regulates apical cell dynamics during corticogenesis

Katrin Gerstmann *et al.*

Corresponding author: Valerie Castellani, valerie.castellani@univ-lyon1.fr;
Katrin Gerstmann, katrin_gerstmann@gmx.de

Sci. Adv. **8**, eabo4552 (2022)
DOI: 10.1126/sciadv.abo4552

This PDF file includes:

Figs. S1 to S5

Figure S1: Sources of Semas, Nrps and Plexins in the embryonic brain

(A) GFP-immunolabeling of Sema3B-GFP ki/ki and control embryonic brains show the presence of Sema3B proteins at the apical border of the telencephalic choroid plexus at E12.5.

(B) Microphotographs illustrating the control of Nrp1 labelling at the choroid plexus apical border by comparative immunostaining using secondary antibody alone.

(C) Anti-Nrp2 immunolabeling of E12.5 and E13.5 embryonic brains and labelling of E13.5 Nrp2 $^{-/-}$ brain sections show Nrp2-labelling specificity. Arrowhead points to the telencephalic choroid plexus, arrow points to the diencephalic ventricular zone and double arrows point to the meninges. Note the enrichment of Sema3B-GFP, Nrp1 and Nrp2 at the apical border of CP epithelial cells.

(D) *In situ* hybridization detects abundant Nrp2 transcripts in the midbrain floor plate at E13.5.

(E) Single cell transcriptome analysis of embryonic cortical cells reveals the expression of Plexins at different embryonic stages (between E12-E15). Pseudotime describes the differentiation from an apical progenitor cell (0) to a postmitotic neuron (1).

(F) Immunolabeling of PlexinB1 and PlexinB2 with different primary antibodies on cortical sections at E12.5 show the expression of these Semaphorin receptors in the developing cortex. Arrows point to the accumulation of the Plexin proteins at the apical surface.

Scale bars: 100µm.

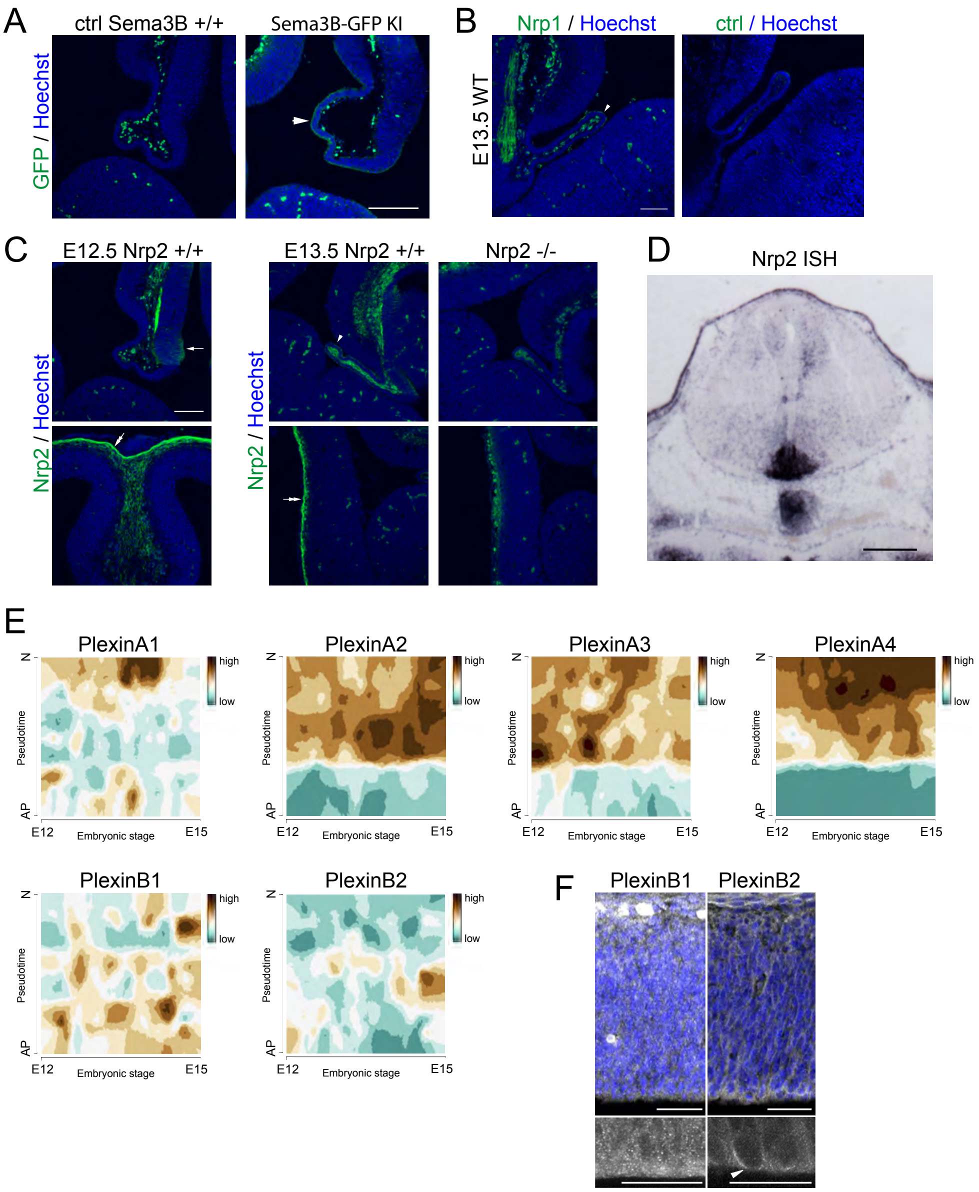

Figure S2: CSF-derived Sema/Nrp-complexes are dispensable for progenitor division orientation and neurosphere formation.

(A) E12.5 cortical sections of WT, Sema3F -/-, Nrp1$^{Sema/Sema}$, Sema3B -/- and Nrp2 -/- embryos were stained with antibodies against phospho-histone 3 (PH3) and γTubulin and the division orientation of cells in ana-/telophase with respect to the apico-basal axis was examined according to [12]. Angle distributions are represented in distinct classes (90°-80°, 80°-60°, 60°-30°,30°-0) in **(B)** pie charts and as cumulative fraction curves. No significant difference in the orientation of the division plane was observed. n= number of analysed cells (3 embryos).

(C) Recombinant Sema/Nrp-Fc proteins do not affect the formation of neurospheres. Mean ± SEM; each dot represents one neurosphere (2 experiments).

(D) Quantification of cell cycle phases of PH3-positive nuclei after intraventricular injection (n= 2 embryos) of recombinant control-Fc, Sema3F/Nrp1-Fc, or Sema3B/Nrp2-Fc (left) and in the mutant mouse lines (n=3 embryos).

Scale bars: A 5 µm, B 100 µm.

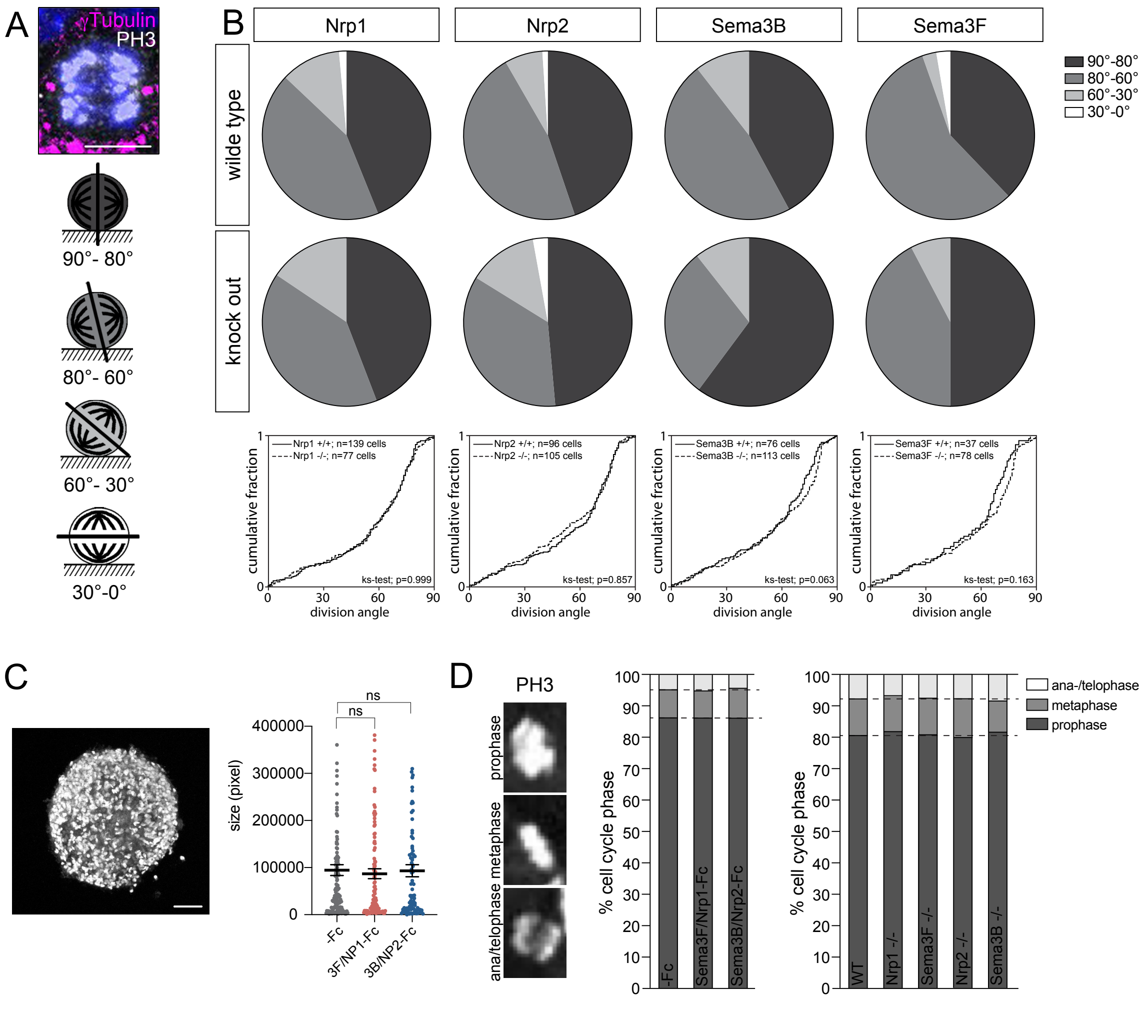

Figure S3: The number of Sox2 and PH3-positive cells are not altered in the absence of CSF-derived Sema/Nrp-molecules.

E12.5 cortical sections of WT, Sema3F -/-, Nrp1$^{Sema/Sema}$, Sema3B -/- and Nrp2 -/- embryos were stained with antibodies against **(A)** Sox2 (sex-determining region Y-box containing gen 2) and **(B)** phospho-histone 3 (PH3) and positive nuclei were counted. No significant changes were observed.

(C) Immunolabelling of Sema3B/3F double mutant embryos against Sox2, PH3, and Tbr1 do not reveal significant changes in comparison to wildtype littermates. However, the numbers of Tbr2-positive cells are reduced in Sema3B/3F knockout mice. This effect disappears at later stages (**D**). **(E)** Sox2-staining at E13.5 reveal no differences in Nrp1$^{Sema/Sema}$ and Nrp2 -/- embryos.

Scale bars: 25 µm. Mean ± SEM; each dot represents one embryo; paired t-test, ** p<0.01, *** p<0.001.

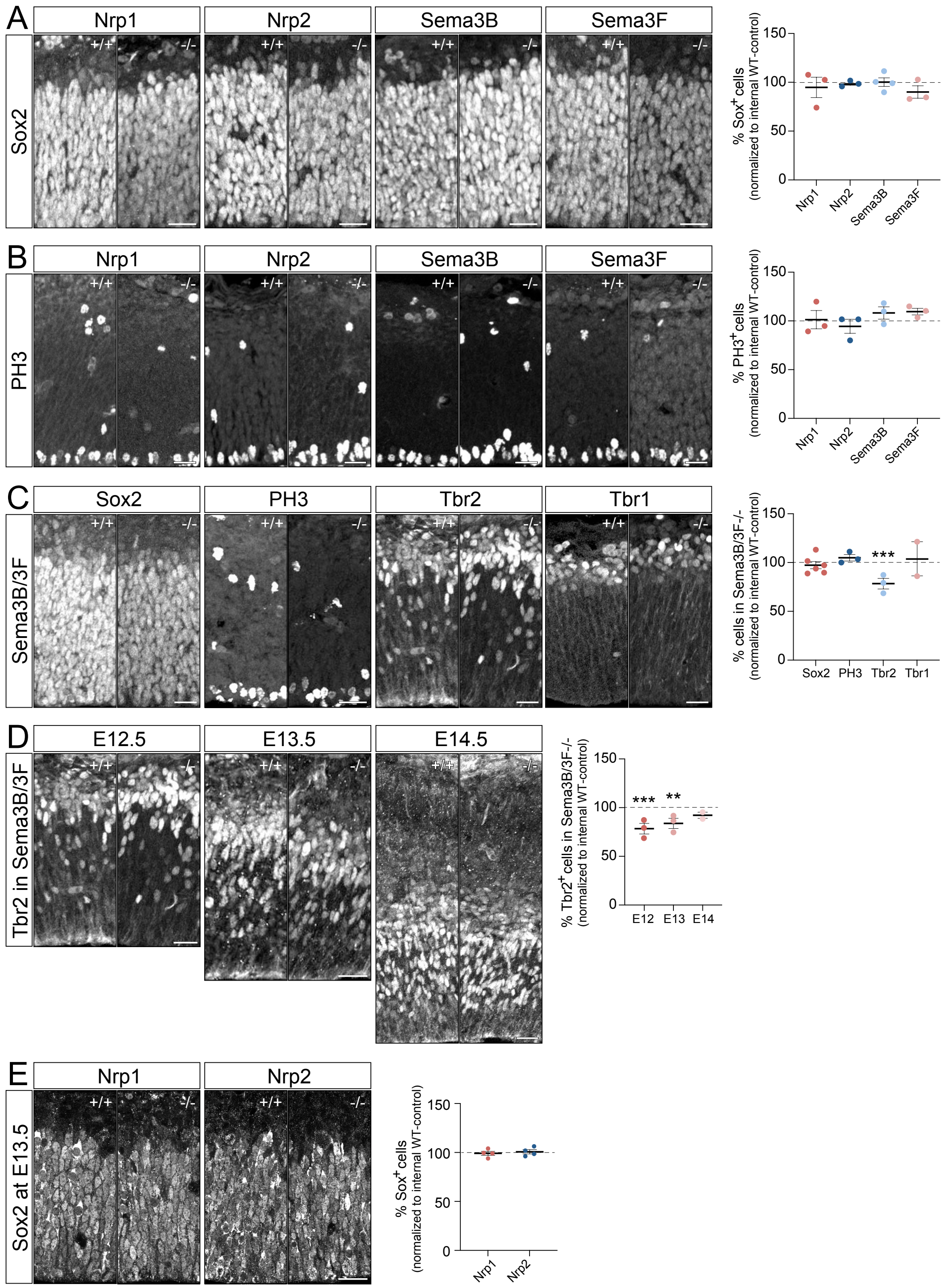

Figure S4: Loss of Nrp1 or Nrp2 function do not alter N-cadherin or β-Catenin at the adherens junction of apical mitotic cells.

Immunostaining of N-cadherin **(A)** or β-Catenin **(B)** on E12.5 cortical sections of Nrp1 and Nrp2 mutant mice. Images show the signal intensity along the apical border of the ventricular zone. Arrows point to adherens junctions of apical mitotic cells. The quantification shows the signal intensity along the ventricular surface (left) or specifically the adherens junctions in apical mitotic cells (right).

Scale bar: 10 µm. Mean ± SEM; each dot represents one slice (intensity along the apical border) or a RGC (mitotic cell intensity); Nrp1 +/+ n=3 embryos, Nrp1 -/- n=2 embryos, Nrp2 +/+ n=2 embryos, Nrp2 -/- n=4 embryos; ks-test, * $p<0.05$.

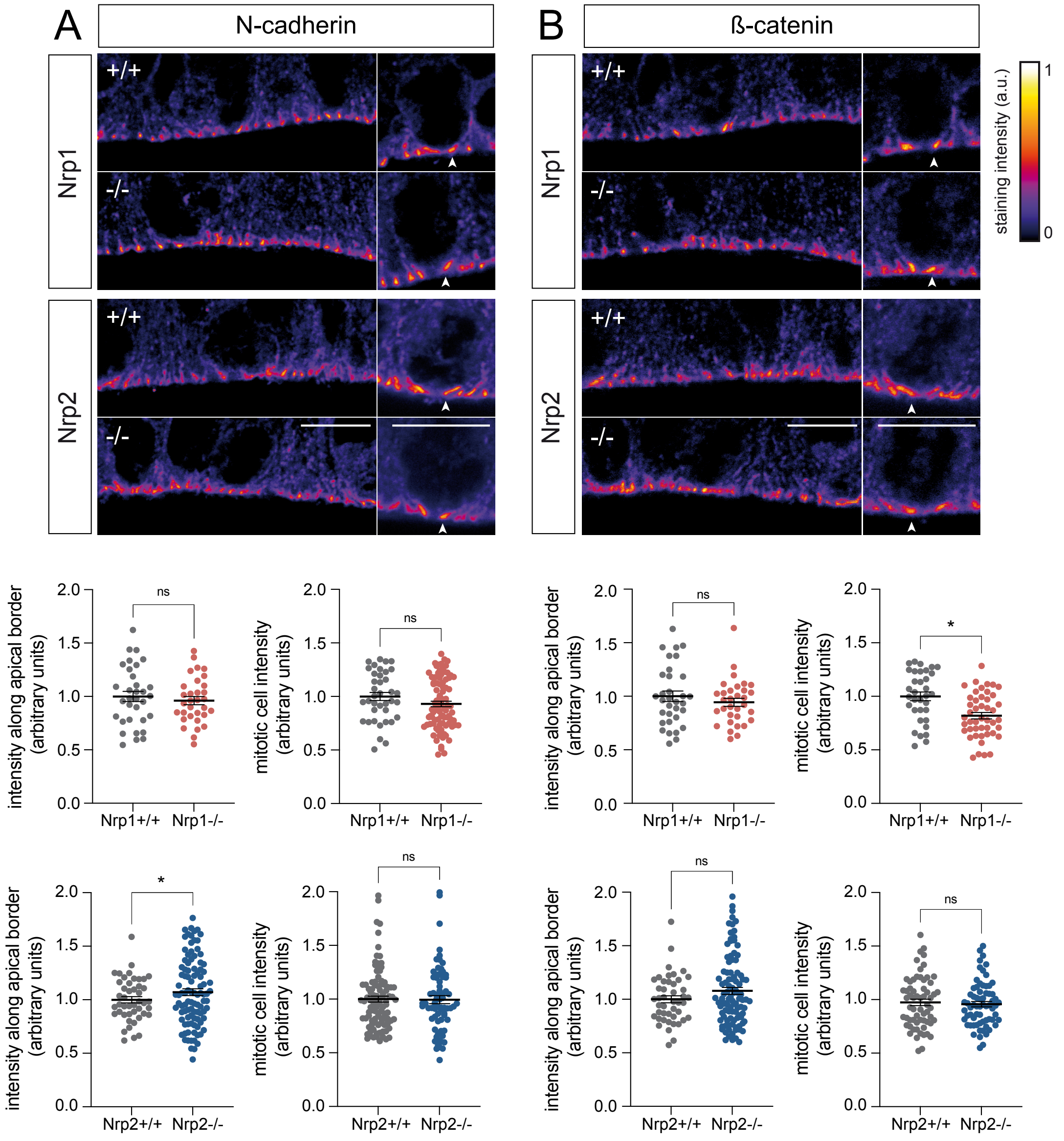

Figure S5: GSK3 differentially modulate Sema3s impact on mitotic nuclei positioning in apical progenitors.
(A) Phalloidin-FITC and Hoechst staining on cortical sections from E12.5 mouse brains submitted to intraventricular exposure of Sema3B/Nrp2-Fc, Sema3F/Nrp1-Fc or ctrl-Fc with or without SB216763 GSK3 inhibitor. Images show the apical surface.
(B) Quantification of the distance of RGC mitotic nuclei from the apical surface, normalized to ctrl-Fc in indicated conditions.
Scale bar: 10 µm. Mean ± SEM; each dot represents one RGC (n=2 embryos), ks-test, *** p<0.001.

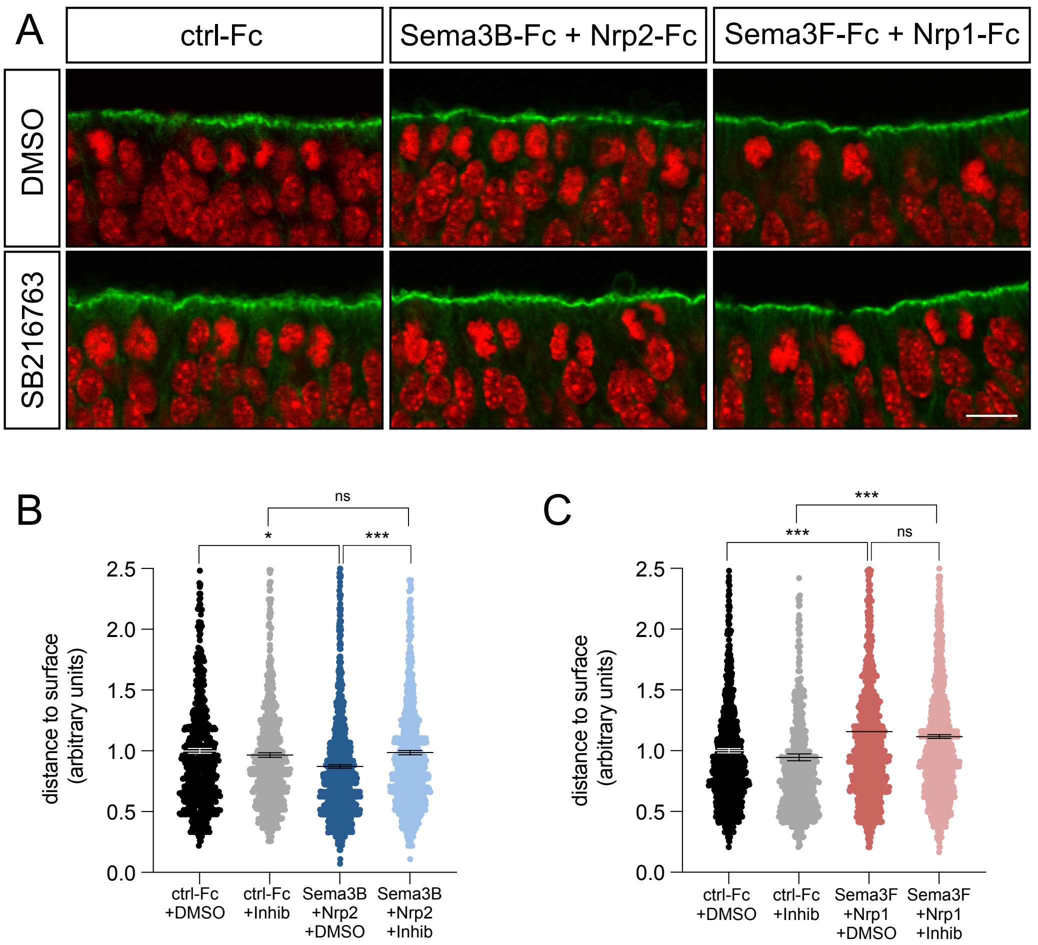

Supplement: Supplementary file 1 — Figs. S1 to S5 [file sciadv.abo4552_sm.pdf]
